# Clinic-Based Delivery of the Young Men’s Health Project (YMHP) Targeting HIV Risk Reduction and Substance Use Among Young Men Who Have Sex with Men: Protocol for a Type 2, Hybrid Implementation-Effectiveness Trial

**DOI:** 10.2196/11184

**Published:** 2019-05-21

**Authors:** Jeffrey T Parsons, Tyrel Starks, Sitaji Gurung, Demetria Cain, Jonathan Marmo, Sylvie Naar

**Affiliations:** 1 Center for HIV Educational Studies and Training Hunter College City University of New York New York, NY United States; 2 Hunter Department of Psychology Hunter College City University of New York New York, NY United States; 3 Health Psychology and Clinical Science Doctoral Program Graduate Center City University of New York New York, NY United States; 4 College of Medicine Florida State University Tallahassee, FL United States

**Keywords:** HIV, motivational interviewing, men who have sex with men, adolescents, comparative effectiveness research

## Abstract

**Background:**

Young men who have sex with men (YMSM) are disproportionately at risk for HIV and sexually transmitted infections. Adapting and testing the effectiveness of the Young Men’s Health Project (YMHP), an efficacious intervention designed to reduce substance use and condomless anal sex (CAS) among YMSM, at clinics in Miami, Detroit, and Philadelphia has the potential to reduce HIV and STI disparities among urban YMSM.

**Objective:**

This study (Adolescent Medicine Trials Network for HIV/AIDS Interventions [ATN] 145 YMHP) aims to adapt YMHP for clinic and remote delivery by existing clinic staff and compare their effectiveness in real-world adolescent HIV clinics. This protocol is part of the ATN *Scale It Up* program described in a recently published article by Naar et al.

**Methods:**

This is a comparative effectiveness hybrid type-2 trial of the YMHP intervention with 2 delivery formats—clinic-based versus remote delivery—offered following HIV counseling and testing. Phase 1 includes conducting focus groups with youth to obtain implementation feedback about the delivery of the YMHP intervention and intervention components to ensure culturally competent, feasible, and scalable implementation. Phase 2 includes recruitment and enrollment of 270 YMSM, aged 15 to 24 years, 90 at each of the 3 sites. Enrollment will be limited to HIV-negative YMSM who report recent substance use and either CAS or a positive STI test result. Participants will be randomized to receive the YMHP intervention either in person or by remote delivery. Both conditions involve completion of the 4 YMHP sessions and the delivery of pre-exposure prophylaxis information and navigation services. A minimum of 2 community health workers (CHWs) will be trained to deliver the intervention sessions at each site. Sessions will be audio-recorded for Motivational Interviewing Treatment Integrity (MITI) fidelity coding, and CHWs and supervisors will be given implementation support throughout the study period.

**Results:**

Phase 1 focus groups were completed in July 2017 (n=25). Feedback from these focus groups at the 3 sites informed adaptations to the YMHP intervention manual, implementation of the intervention, and recruitment plans for phase 2. Baseline enrollment for phase 2 began in November 2018, and assessments will be at immediate posttest (IP)-, 3-, 6-, 9-, and 12-months after the intervention. Upon collection of both baseline and follow-up data, we will compare the effectiveness and cost-effectiveness of clinic-based versus remote delivery of YMHP in the context of health care access.

**Conclusions:**

We are conducting YMHP in 3 cities with high rates of YMSM at risk for HIV and STIs. When adapted for real-world clinics, this study will help substance-using YMSM at risk for HIV and STIs and allow us to examine differences in effectiveness and cost by the method of delivery.

**Trial Registration:**

ClinicalTrials.gov NCT03488914; https://clinicaltrials.gov/ct2/show/NCT03488914 (Archived by WebCite at http://www.webcitation.org/770WaWWfi)

**International Registered Report Identifier (IRRID):**

DERR1-10.2196/11184

## Introduction

### Background

Young men who have sex with men (YMSM; aged 16-24 years) are disproportionately at risk for HIV and sexually transmitted infections (STIs). Although new HIV infections have fallen or remained stable among other groups, YMSM have experienced a 132% increase in new infections since 2002 [1]. From 2008 to 2011, YMSM aged 13 to 24 years had the greatest percentage increase (22%) in new HIV infections [2]. YMSM of color are especially at risk: in 2011, among YMSM aged 13 to 24 years with HIV infection, 58% were black and 20% were Latino [2].

Young males aged 15 to 24 years are vastly overrepresented in rates of STIs. In 2013, males aged 15 to 24 years accounted for more than half (57%) of all male cases of Chlamydia trachomatis (CT) infection and 46% of Neisseria gonorrhea (GC) [3]. Young men aged 20 to 24 years had the highest rate of syphilis from 2008 to 2012 [3]. Furthermore, sexual orientation disparities exist—for example, men who have sex with men (MSM) accounted for 75% of all syphilis cases in 2013 [3]. Ethnic and racial disparities exist in the incidence of other STIs among YMSM [3]. Young black men aged 15 to 24 years have a rate of CT infection 5.5-9.5 times higher and GC rates 10.4-13.0 times higher than white men [3]. A recent study of HIV-negative MSM diagnosed with rectal CT or GC at STI clinics between 2008 and 2010 showed that such infections greatly increase HIV incidence [4]. In 2013, MSM accounted for 3 quarters of all primary and secondary syphilis cases diagnosed in the United States—an increase of 10% since 2012 [3].

Location is also a risk factor. YMSM in large urban areas are disproportionally affected. Detroit, Philadelphia, and Miami have high rates of HIV and STIs among males. These cities are geographically diverse and have large numbers of YMSM receiving HIV counseling and testing annually by clinic sites and community partners. This information points to the need to study the effectiveness of the Young Men’s Health Project (YMHP), for its significant public health potential in reducing HIV/STI disparities among urban YMSM.

### Evidence Base

#### Substance Use and HIV Risk Among Young Men Who Have Sex With Men

MSM use substances at higher rates than the general population, increasing HIV risk. High rates of drug and alcohol use among MSM relative to the general population have been documented [5-7], and our research has identified higher rates of drug use among YMSM compared with heterosexuals [8-10]. Higher rates of drug use have also been documented among MSM, including YMSM in tandem with sexual activity [11,12]. However, drug-use patterns seem to differ among YMSM with increased rates of cocaine among YMSM, which could have implications for HIV risk given that stimulant use has been linked to condomless anal sex (CAS) [13-16] and higher risk of HIV infection and other STIs [17]. Nearly half of black MSM with newly diagnosed HIV infection (48%) reported substance use during their last anal sex encounter [11]. Substance use has been found to increase sexual risk behavior among MSM [18], placing them at high risk for CAS and HIV seroconversion, exchange sex, and greater number of sexual partners [19]. Our own research using event-level data for the previous 30 days has shown that substance use strongly and significantly predicts the odds of whether YMSM will use a condom [20]. A number of other studies have looked at the impact of substance use on sexual behavior and increased odds of seroconversion [11]. Different substances are associated with sexual risk behavior among specific groups. Among Latino MSM, methamphetamine use [21,22] and among black MSM higher rates of marijuana use have been linked to sexual risk [23]. Therefore, there is a critical need for brief, culturally appropriate, effective behavioral interventions that improve self-management to reduce new HIV infections among substance-using YMSM.

#### Effectiveness of Motivational Interviewing

Motivational interviewing (MI) has the potential to improve self-management behaviors in terms of promoting sexual health and reducing substance use among YMSM. There is strong evidence that MI is a culturally appropriate and effective approach for working with racial and ethnic minority populations [24] who are disproportionately affected by HIV. One meta-analysis of MI found a greater effect among minorities [25]. MI has been recommended as particularly effective when working with YMSM [26]. MI promotes increased intrinsic motivation to change and, when paired with information regarding health risk behaviors, reinforces the individuals’ right and capacity to make well-informed health self-management decisions for themselves [27].

YMHP, a manualized structured 4-session intervention using MI and problem-solving skills building, has been listed as a best evidence intervention by the Center for Disease Control and Prevention (CDC) and is the only intervention to reduce both CAS and substance use among YMSM [28]. The CDC’s endorsement of YMHP was informed by results of the National Institutes of Health (NIH)-funded efficacy trial. A total of 143 HIV-negative YMSM (aged 18-29 years) who reported CAS with a high-risk partner and at least 5 days of drug use in the last 3 months were randomized to receive 4 sessions of YMHP or 4 sessions of health education on sexual risk and substance use. The majority [63%(90/145)] of the sample were YMSM of color (black: 21%, Latino: 28.7%, or multiracial: 13.3%). Retention rates were high: 79% at the 12-month follow-up and 88% completed at least 3 of the 4 sessions. Master’s level therapists were trained in MI with ongoing fidelity monitoring using the Motivational Interviewing Treatment Integrity (MITI) coding system. YMSM who received YMHP reduced their cross-time averaged odds of ever using any drug by 67% over the 1-year follow-up. Within-condition analyses showed that YMSM who received YMHP reduced their cross-time averaged odds of ever having CAS by 83% over the 1-year follow-up. YMSM in the YMHP condition were 21% less likely to report CAS on days when they did report drug use relative to men receiving education. This has been the first and only RCT with HIV-negative YMSM to show significant reductions in both CAS and substance use. As such, YMHP has the potential to have a significant impact on YMSM seeking sexual health or HIV counseling and testing services at clinics.

#### Remote Delivery

Research on telephone-based MI has consistently found that it produces significant improvements in a wide range of physical health challenges [29-31]. Furthermore, telephone-based MI reduces mental health–related problems [32] and alcohol-related problems [33] as well as sexual risk taking [31,34-36] among HIV-positive individuals. Research comparing the effects of telephone-based MI with face-to-face (clinic-delivered) interventions has produced equivocal results. Across studies examining physical activity, mental health, and substance use outcomes, findings suggest no significant differences in delivery method [34-37]. Carey et al examined the relative efficacy of a telephone versus face-to-face intervention for alcohol use and observed a significant interaction of delivery method with gender [38]. Women on average had better outcome in the face-to-face condition, whereas men responded equally well to both delivery methods.

Notably, the issue of health care access has not yet been examined as a moderator of relative effectiveness. One advantage of telephone-based MI is that it significantly reduces patient burden. It is, therefore, plausible that it will show superior effects among YMSM who experience barriers to health care access. However, it is also possible that remote delivery (via phone or video chat using Skype or FaceTime) will decrease engagement and the quality of the relationship between the clinicians and the participant. This might result in clinic-based delivery being superior among YMSM who have better access to health care. Understanding how health care access intersects with delivery method will substantially inform implementation decisions at clinics and other agencies seeking to utilize YMHP.

#### Community Health Workers Intervention Delivery

Integrating implementation science into a comparative effectiveness trial (CET) can minimize the science-practice gap [39]. MI providers need not be clinicians—1 study conducted by our team comparing community health workers (CHWs) with clinicians found both were equally effective in providing high-quality MI and that clients were more likely to be retained in HIV care when working with CHWs [40]. CHWs are commonly integrated into clinics and often play a central role in providing HIV prevention services, including HIV counseling and testing. Training CHWs to deliver evidence-based interventions is a critical step toward realistic and cost-effective implementation [41]. CDC has called for expanded use of CHWs in services for chronic disease [42] with attention to implementation and training [43-50].

Several steps can be taken in a CET of YMHP to promote adoption and sustainability. First, using staff embedded in the existing clinic setting can build capacity for implementation. Second, CHWs have long been the cornerstone of integrating support services into HIV-related prevention efforts [51]. Training CHWs to deliver interventions is a critical step toward realistic and cost-effective implementation. Research has documented the amount of training needed to obtain MI fidelity, concluding that initial training followed by ongoing coaching is required [52-55]. Such training can be costly when relying on outside trainers. Thus, a *train the trainer* model where expert trainers provide local supervisors with MI coaching skills might be more sustainable [56]. Finally, CET designs help gain information about implementation [57]. In a *Hybrid 2* trial, the goal is to dually determine which treatments work in which settings and to simultaneously answer implementation science questions about the potential barriers/facilitators to a treatment’s widespread and continued implementation.

### Aims

The aim of this paper is to describe Adolescent Medicine Trials Network for HIV/AIDS Interventions (ATN) 145 YMHP to study the scale-up of evidence-based practices in multidisciplinary adolescent HIV care settings while balancing flexibility and fidelity. The protocol is part of the *Scale it Up* research program focusing on implementation of self-management interventions to impact the adolescent HIV prevention and care cascades [58]. The purpose of this study is to adapt YMHP for clinic and remote delivery by existing HIV clinic staff, CHWs, who work with YMSM aged 15 to 24 years. This study also aims to (1) compare the effectiveness of clinic-based versus remote delivery of YMHP in the context of health care access by hypothesizing that remote-delivery will yield significantly better results among youth who experience barriers to health care access, whereas clinic-based delivery will yield significantly better results among youth who do not, (2) assess the cost-effectiveness of both formats of delivery to increase the likelihood of uptake for this intervention, and (3) assess the 5 components of the self-management model (ie, problem solving, decision making, resource utilization, forming of a patient and health care provider partnership, and taking action) and how these components vary over time, are directly improved by the interventions, and mediate intervention effects. Finally, a sustainable model of YMHP will be tested in real-world adolescent clinics utilizing the *Exploration, Preparation, Implementation, Sustainment model (EPIS;* see ATN 153 EPIS protocol paper [59]).

## Methods

### Overview of Content and Delivery

This is a comparative effectiveness hybrid type-2 trial of the YMHP intervention designed to achieve aims over 2 phases. Phase 1 was completed by conducting focus groups with youth as part of its formative research to obtain implementation feedback about the delivery of the YMHP intervention and intervention components to ensure culturally competent, feasible, and scalable implementation both in the clinic setting and via remote condition, when delivered by CHWs. For phase 2, the target sample size is 270 YMSM, aged 15 to 24 years, 90 at each of the 3 sites. Participants are randomized at the end of their baseline assessment into 1 of the 2 intervention conditions: (1) delivery of the YMHP intervention in person at the clinic site or (2) delivery of the YMHP intervention by phone or video chat using apps such as Skype or FaceTime. Everyone will receive session 1 in person immediately upon completion of the baseline assessment regardless of their randomization to the 2 intervention conditions. Those randomized to face-to-face clinic-based delivery will schedule and complete their remaining 3 sessions in person, whereas those randomized to the remote condition will schedule and complete the remaining 3 sessions remotely.

**Figure 1:**
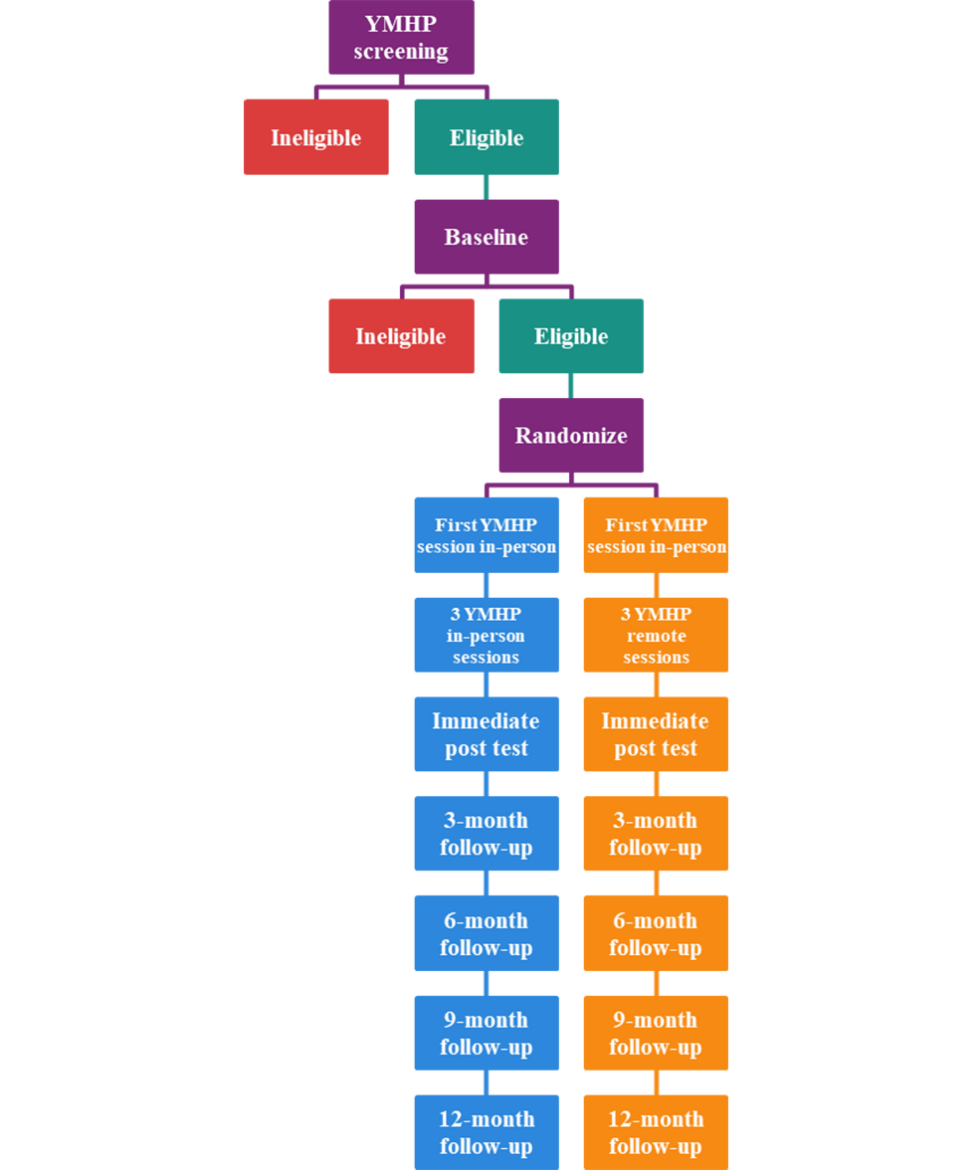
Participant flow diagram. YMHP: Young Men’s Health Project.

The study will employ a stratified randomization procedure based on city, minority status, and health care access, so that within each city, youth who experience barriers to health care access are distributed equally across conditions. We will also stratify by interventionist and whether a youth has used marijuana only versus other drugs in the past 3 months. Research staff will complete a survey on Qualtrics that will randomize participants into 1 of the 2 study groups with no masking to the research staff or participant. Intervention sessions will occur approximately once per week for 4 weeks beginning 1 week after completion of the baseline assessment. There is a 12-week window for intervention completion, that is, all 4 sessions must be completed within 12 weeks of the baseline assessment. The immediate posttest (IP) assessment will occur 3 months after baseline and subsequent follow-ups will be scheduled 3 months thereafter until the 12-month postintervention (ie, 15 months postbaseline) assessment. Baseline, 3-month, and 9-month assessments consist of self-report data collection (CASI) and HIV and STI testing for CT, GC, and syphilis. The IP, 6-month, and 12-month follow-up assessments require completing the CASI only and can be completed remotely via email Qualtrics link. All enrolled participants could receive up to US $275 by the end of this study—US $50 for baseline, which includes CASI, bio-testing, and session 1; US $30 for sessions 2 to 4; US $20 as a bonus for completing all 4 sessions; US $25 for CASI-only assessments (ie, for IP, 6-month, and 12-month assessments); and US $50 for assessments with both CASI and bio-testing (ie, for 3-month and 9-month assessments). The participant flow diagram is presented in Figure 1.

### Recruitment and Eligibility

#### Recruitment

**Figure 2:**
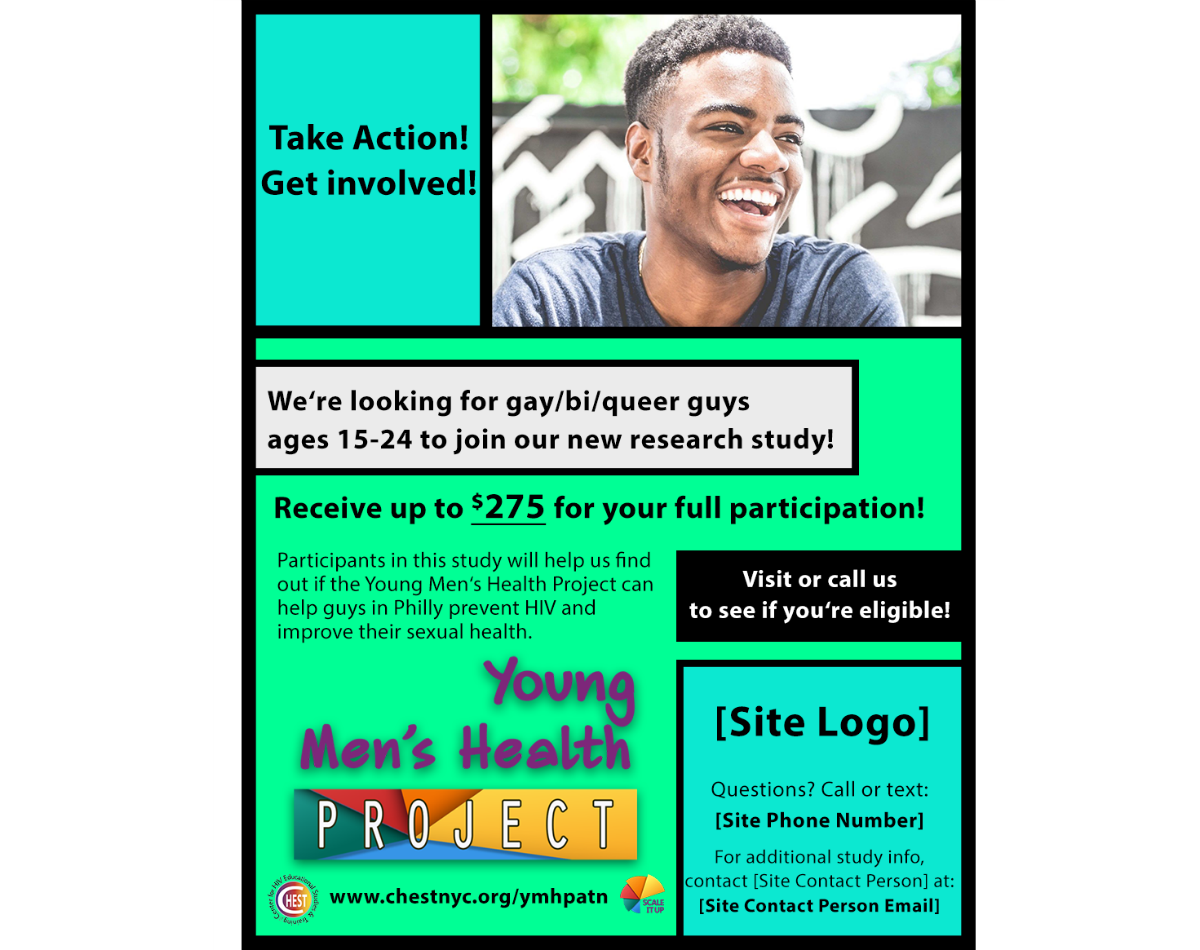
Young Men’s Health Project recruitment flyer.

The Center for HIV Educational Studies and Training (CHEST) at Hunter College will use a variety of recruitment strategies to recruit participants for this study. First, as a result of CHEST’s role as the Management Core and Recruitment and Retention Center (REC) for *Scale It Up*, we will utilize 3 Subject Recruitment Venues (SRVs) in our network (Wayne State University Prevention in Detroit, University of Miami, and Children’s Hospital of Philadelphia) to complete clinic- and field-based recruitment. All 3 SRVs, as well as CHEST, have extensive relations with the gay, lesbian, bisexual, and transgender communities; community service organizations; health service organizations; and providers for MSM. In this aspect, recruitment will occur from routine walk-in visits for HIV testing. Partnering clinics will include offering this study through outreach activities and through their HIV testing services they provide in their clinics. Information about the study will be included in the institutional review board (IRB)-approved palm cards, brochures, and flyers at each clinic site (see Figure 2). This information will be displayed in waiting rooms and exam rooms at the clinic as well as at locations of mobile testing events and outreach shifts to be passed to potential participants to both encourage HIV testing and promote the YMHP study. This method has worked well in the past for numerous studies and takes advantage of when patients have more idle time to learn about the study.

YMSM who test HIV negative at the 3 SRVs in Miami, Detroit, and Philadelphia or through mobile testing efforts provided by community collaborators will be offered the opportunity to participate in YMHP. Those interested will be asked to complete a brief *Study Screener* on a study iPad to collect demographic and behavior questions related to eligibility criteria. Participant ID numbers are generated and assigned through Qualtrics during the screening process to all potential participants, including those who screen ineligible. If eligible, a screen will be displayed informing the potential participant of his eligibility, and the site study staff person will then schedule the potential participant for a baseline assessment. We anticipate enrollment of 5 participants per month, per SRV, and have allocated staffing resources to ensure this rate.

In addition, CHEST will assist in referring potentially eligible participants to the YMHP study through existing Web-based recruitment efforts. CHEST utilizes the Hunter College IRB-approved online master screener (OMS) used to preliminarily screen individuals who are interested in participating in studies being conducted through CHEST. If an individual is preliminarily eligible for a study, the individual is asked to provide contact information to CHEST for follow-up. For the purposes of this study, the OMS will be used to refer potentially eligible YMSM to HIV testing sites by sending them an email referral informing them about the YMHP study and where to go to determine eligibility after completing the OMS. Potential participants will also be called by CHEST staff to complete the *Study Screener* over the phone. If they meet the study criterion, they will be scheduled by the study staff to attend the clinic for HIV testing and YMHP enrollment. The OMS, in this instance, will primarily be used as a referral mechanism for the study, directing participants to which study they might be eligible for, including YMHP.

#### Eligibility Criteria

All interested participants are assessed for eligibility by completing a brief *Study Screener*. Study inclusion criteria include (1) being aged between 15 and 24 years; (2) currently identifying as male (regardless of birth sex); (3) receiving an HIV-negative test result from a study site or mobile HIV testing in the past 90 days; (4) having sex with men in the past 90 days; (5) reporting at least 3 days of illicit drug use or heavy drinking (5 or more drinks) in the past 90 days; (6) reporting at least one episode of CAS with a male partner in the past 90 days or a positive STI test result in the past 90 days; (7) living in the Detroit, Miami, or Philadelphia metropolitan areas; and (8) having the ability to communicate in English. Exclusionary criteria include the following: (1) participants whose mental, physical, or emotional capacity does not permit them to complete the protocol as written; (2) currently taking Truvada as pre-exposure prophylaxis (PrEP); and (3) 5 or more days of injection drug use in the past 90 days.

### Management and Tracking Study Visits

There are 2 different types of visits during the study timeline. Research visits include all assessments including the baseline visit, the posttest assessment, and all other follow-up assessments. All participants, in either condition, are expected to complete 6 research visits as part of full participation in the study. Research visits will be tracked using REDCap, a secure Web app for managing Web-based surveys and databases. This system allows both SRV study staff and the REC staff to monitor the completion of study visits and surveys and generate reports on enrollment and retention as needed. SRV study staff will track all completed study components in REDCap immediately after completing an assessment no later than the end of the business day. Intervention visits include all YMHP sessions between enrolled study participants and trained CHWs. Intervention visits will be largely managed by the CHWs with their individual study participants. CHWs will notify SRV study staff of whether visits were completed for tracking purposes, provide receipts for session compensation to SRV study staff, and upload audio files to Dropbox Business as per study procedures. CHWs will deliver the first YHMP session at the completion of each baseline assessments immediately, regardless of randomization, and will schedule the subsequent session in person or remotely.

### Intervention Design

YMHP has been listed as a best evidence intervention by the CDC and is the only intervention to show significant effects on both CAS and substance use among YMSM. MI techniques are utilized to provide personalized feedback for reducing CAS and substance use among YMSM. The YMHP intervention will be delivered by MI-trained CHWs employed at SRVs, primarily by former HIV counseling and testing counselors, health educators, and trained program peers. Participants will be randomized to receive the intervention either in person or remotely by phone or via video chat using Skype or FaceTime. Both conditions involve completion of the 4 YMHP sessions and the delivery of PrEP information and navigation services to interested participants.

### Four Sessions of Young Men’s Health Project

In session 1, youth will choose which behavior to discuss first (sexual risk or substance use), and the CHW will elicit the participant’s view of the problem using standard MI techniques, building motivation for change by eliciting and reinforcing change talk and clarifying the youth’s own personal priorities (through a structured card sorting activity). The CHW will assess and reflect the participant’s readiness to make changes in target behavior. If the participant is willing to proceed, goals on healthy behaviors (ie, substance moderation, sex risk harm reduction strategies) are set. The session ends with MI strategies to evoke the youth’s ideas about how to take steps toward change, consolidate the youth’s commitment to the plan, and problem solving. Session 2 follows the same format as session 1 but revolves around the second target behavior. Session 3 includes a discussion about how PrEP might fit within a youth’s goal for healthy behaviors. In sessions 3 and 4, the CHW will review the change plan, continue to elicit and reinforce change talk, problem-solve barriers, consolidate commitment, and address maintenance of behavior change.

### Training of Interventionists

YMHP intervention training will occur before the initiation of phase 2, with ongoing coaching and supervision and training of new interventionists, as required. The interventionist training team includes 2 members of the Motivational Interviewing Network of Trainers from the CHEST, and this training procedure includes (1) an initial 3-day training for CHWs and local supervisors; (2) a 2- to 3-month training period of role-play practice, coding and feedback, and supervision modeling, including mock sessions with *standardized participants* role played by CHEST research assistants; (3) 1-hour weekly supervision sessions between local supervisors and CHWs; (4) monthly supervision calls between local supervisors, the interventionist training team, and the protocol lead including a quarterly Skype booster training; and (5) ongoing quality assurance and feedback using MITI coding.

All materials (eg, slides, training exercises, supervisory tools) will be packaged for potential dissemination. Before dissemination, any copyrighted media will be removed from these materials. The initial 3-day training was held for all CHWs and their supervisors in Miami and followed a curriculum developed from previous NIH-funded effectiveness trials. CHWs and supervisors participated together in days 1 and 2 of the training. The third day of training was split so CHWs could have more practice with the YMHP protocol and supervisors could focus on coaching MI. CHEST provided external MITI coding for the supervisors to use as feedback. Following the 3-day training workshop, all CHWs and supervisors submitted audio recordings of all intervention sessions. Mock sessions are completed as in-person and remote delivery with mock participants. These sessions were MITI coded and CHEST trainers provided coaching and feedback. Once beginner competency was met, the local SRV supervisor took over weekly individual supervision of the CHWs. Throughout YMHP, the interventionist training team will continue to provide support to supervisors for assistance in supervision and will focus on having them practice listening for skills and then model for them how to use MITI feedback.

### Fidelity Monitoring

All sessions (clinic-based and remote-based) will be audio-recorded, and one recording per CHW will be randomly selected for MITI coding by the research team on a regular basis. For the full trial, a random selection of 10% of the interviews will be independently coded. Supervisors will complete fidelity checklists for the supervision session so the team can monitor implementation. The protocol lead and the interventionist training team will facilitate quarterly boosters via group Skype for supervisors. Before the quarterly boosters, supervisors will submit a recording of a supervision session for review. Boosters will cover successes and challenges, MITI scores, updated MI skill development plans for each CHW, and role-plays of supervision skills. The protocol lead and the interventionist training team will join supervision sessions via Skype if MITI scores fall below competency without remediation. They will also lead annual in-person booster trainings covering MI skills and specific delivery of YMHP for supervisors and CHWs. All boosters will be recorded and qualitatively analyzed.

## Results

### Phase 1

Phase 1 was conducted in 2017, with site visits to Miami, Detroit, and Philadelphia where focus groups were conducted with youth. Focus groups participation breakdown is provided in Table 1. The feedback from the focus groups at each clinic has been used to modify the YMHP intervention before the launch of phase 2. Phase 1 focus groups were conducted with a total of 25 youth across the 3 SRVs to gather information that would be used to better implement YMHP. Youth were divided into 2 age groups, 15 to 17 years and 18 to 24 years. The first focus group was conducted in Detroit between June 7th and 9th of 2017 with 14 scheduled potential participants. Out of those, 7 participants provided consent and participated in the group discussion. The second focus group was conducted in Philadelphia between July 10^th^ and 11^th^ with 23 scheduled potential participants. Out of those, 8 participants provided consent and participated in the group discussion. The final group was conducted in Miami between July 12^th^ and 14^th^ with 20 scheduled potential participants. Out of those, 10 participants provided consent and participated in the group discussion. A total of 25 participants who reported their HIV status as negative attended focus groups and provided their feedback on the YMHP intervention.

Participants in focus groups were asked to provide feedback on HIV testing and counseling experiences and how to incorporate screening into the testing process, the YMHP intervention, and barriers to completing participation in the study. Participants reported positive experience working with the 3 sites and limited negative experiences overall with testing and counseling. Many participants expressed a lack of discussion about substance use but a desire to engage in discussions about it with CHWs, especially in the context of peer pressure from older partners. Participants at all SRVs reported cocaine and ecstasy as commonly used substances in their cities. When participants were asked about the intervention, they expressed interest in the remote delivery option. Many thought that the advantages to sessions over the phone or video chat such as Skype and FaceTime are that it eliminates transportation as a barrier to session completion and the resistance of talking to a therapist face-to-face. Participants also felt that individual characteristics (eg, race, gender) of the CHW delivering the YMHP intervention did not matter as long as they were confident with their knowledge and the resources they are offering. In addition, they perceived scheduling and discretion were the 2 biggest barriers to completing sessions because of school and needing parents to possibly transport them to and from appointments at the SRV, while also having to explain the study to strict parents for youth less than or equal to 18 years as an obstacle.

**Table 1:** Young Men’s Health Project focus groups.

Site	Date	Participants	
		Age group 15-17 years (n=3)	Age group 18-24 years (n=22)
Detroit	June 7-9, 2017	0	7
Philadelphia	July 10-11, 2017	2	6
Miami	July 12-14, 2017	1	9

### Phase 2

On the basis of the results from phase 1, for phase 2 we will enroll 270 YMSM, with 90 participants per site and 135 participants in each of the 2 conditions. Recruitment for phase 2 began in October 2018, and all participant components are projected to end in December 2020.

#### Intervention Outcomes and Measures

This study examines 4 coprimary outcomes related to substance use (1 outcome) and sexual health management (3 outcomes). These are measured during baseline; immediate postintervention; and 3-, 6-, 9-, and 12-months follow-up assessments. Specifically, sexual health management is measured by 3 outcomes: (1) decreased STIs, (2) decreased CAS, and (3) increased PrEP uptake/adherence. HIV/STI testing occurs after immediate postintervention at 3- and 9-month follow-up assessments.

Substance use is measured using the *Alcohol, Smoking and Substance Involvement Screening Test* (ASSIST) [60]. ASSIST is often utilized as a screener in primary care settings for substance abuse. Specifically, ASSIST assesses participants’ use of alcohol, tobacco, cannabis, sedative, hallucinogens, inhalants, opioids, and other drugs. The primary substance use outcome will be substance use days during the past 90 days.

To measure CAS, participants complete a series of questions pertaining to their sexual behavior with main and casual partners [61]. Participants estimate their total number of sex partners in the past 3 months. Participants also indicate if they had unprotected, receptive or insertive, anal sex with partners who were HIV positive or of unknown HIV status.

The *Motivational PrEP Cascade* is a series of 21 questions that are designed to assess PrEP treatment uptake and adherence [62-64]. Participants report their familiarity with PrEP; experiences and acceptability; as well as PrEP contemplation, preparation, action, and maintenance utilizing the Transtheoretical Model of Change framework. This includes assessing willingness and intentions for PrEP uptake. The *Motivational PrEP Cascade* also assesses when participants begin and stop taking PrEP [65].

#### Putative Moderators and Mediators of Intervention Effects

Moderators and mediators of intervention effects will be assessed with health care access and other self-management constructs for decision making, problem solving, self-regulation, and provider and health care relationship. Health care access is a series of 9 questions adapted from Williams and Chapman’s unmet health and mental health need [66]. Items included whether youth were not able to get health care services (ie, routine physical examination, see a provider for sexual health care or get access to PrEP, psychological or emotional counseling, and counseling for drug or alcohol use) in the past year when they felt they needed those services.

Self-management constructs included the Behavior Rating Inventory of Executive Function (BRIEF-A) [67] and the Patient Activation Measure [68]. BRIEF-A is a series of 22 questions asking patients to rate whether certain problems (eg, bothered by having to deal with changes, do not plan ahead for tasks, and problems completing my work) occurred in the past month. Responses included never, sometimes, and often. For patient activation measure, participants were asked on a 4-point scale, ranging from strongly disagree to strongly agree or not applicable, a series of 10 questions related to their health. Examples of some of the questions include, “When all is said and done, I am the person who is responsible for taking care of my health” and “I am confident that I can follow through on medical treatments I may need to do at home.”

#### Quantitative Analysis Plan

The primary hypothesis is that, although the main effect of YMHP delivery modality will be nonsignificant, there will be a significant interaction between access to health care and delivery modality. Specifically, it is hypothesized that remote-based YMHP will demonstrate greater improvements in sexual health management (as measured by decreased STIs, CAS, and increased PrEP uptake/adherence) as well as greater reductions in substance use, compared with clinic-based YMHP, among YMSM who do report barriers to health care access. In contrast, it is hypothesized that clinic-based YMHP will demonstrate greater improvements in sexual health management and reduced substance use among YMSM who do not report barriers to health care access.

The protocol lead and analytic team will test the effect of clinic-based versus remote-based delivery on STI rates, alcohol and drug use behavior, CAS, and PrEP uptake through multilevel growth mixture modeling (GMM). A separate model will be run for each outcome. Each model will be a 2-level model in which individuals (level I) are nested in clinics (level II). This approach controls for the nonindependence of individuals within clinics. As 3 sites provide extremely limited predictive power at level II, no site covariates are included in the model. The models are empty at level II.

In a GMM, a latent growth curve with an intercept and linear slope factor is specified. Latent class analysis is applied to these 2 growth components (intercept and slope) to identify groups of individuals who share trajectories. For example, with regard to drug or alcohol use, *immediate and sustained responders* might have the lowest postintervention intercept and a flat slope. Meanwhile, *non-responders* might have the highest postintervention intercept and a flat slope. In contrast, *delayed responders* might have a high postintervention intercept but a significant negative slope, indicating reductions in missed medication over the follow-up period.

If modeling results indicate that discrete classes are not present, we will proceed with analyses in which the growth factors (intercept and slope) are predicted directly by intervention condition (and demographic factors found to be associated with condition after randomization or with attrition over follow-up). GMMs can subsequently incorporate predictors of class membership. These analyses can be conceptualized as a multinominal logistic regression with the latent trajectory-class membership constituting the outcome. The predictors of primary interest will be intervention condition, the presence of any barriers to health care access, and the interaction between condition and barriers to access. We will include as covariates any demographic variables that were associated with condition after randomization or with attrition over the follow-up period.

#### Power Analysis and Sample Size

Power analysis was conducted based on the 4 outcomes of STIs, PrEP uptake, alcohol and drug use, and CAS. First, we analyzed power assuming independence of participant observations (assuming that the nesting of people within clinic was irrelevant). With regard to STIs, we utilized the *repeated measures* module of PASS 13.0 [NCSS Statistical Software] to examine power to detect odds ratio differences in a repeated measures design. Specifying compound symmetry, we allowed ρ to vary between .2 and .5. Assuming the prevalence of STIs in remote delivery condition varies between .05 and .15, the proposed design (N=270) has power .80 to detect an odds ratio of 0.20 to 0.50. Similarly, with regard to the odds of PrEP uptake, allowing the rate of uptake in the remote delivery condition to vary between .05 and .20 and allowing ρ to vary between .4 and .7, the proposed design has power of .80 to detect an odds ratio of 1.9 to 2.3. With regard to number of alcohol and drug use days, we utilized the Tests for Two Poisson Means module in PASS 13.0. On the basis of the data from our previous studies, we allowed the rate of heavy drinking in the remote delivery condition to vary between 7 and 9 days during a 30-day assessment. The study is adequately powered to detect a 3% reduction in the number of heavy drinking days in the in-person condition at any single follow-up point. Allowing the rate of substance use in the remote delivery condition to vary between 3 and 5 days during a 30-day assessment period, the study is adequately powered to detect a 4% reduction in drug use in the in-person condition. A similar analysis was conducted with respect to CAS. On the basis of our previous research, we allowed the rate of CAS in the remote delivery condition to vary between 2 and 4 acts in a 30-day assessment period. The study is powered to detect a difference as small as 5% between the remote delivery and in-person YMHP conditions. The nesting of individuals within sites has the potential to reduce power because substantial variability in outcome across sites can obscure level-II treatment effects [69,70]. The design effect can be used to tailor power analyses calculated under assumptions of independence. In the case of a level-I predictor with a fixed effect that is uncorrelated with other covariates in the model, the design effect is equal to the 1−ρ, where ρ is the intraclass correlation or the percentage of variance accounted for by variability between sites. In previous ATN intervention trials, between clinic site variability in HIV-related outcomes did not differ significantly from 0. We anticipate an absence of variability across clinics, suggesting that the design effect would result in a negligible reduction in power. Finally, a sample size of 270 is sufficient to detect a moderation effect with an *f*^2^ of 0.02. Cohen [71] designates this as a small effect; however, recent work has characterized an effect of this size as moderate to large as applied to moderation [72].

#### Cost-Effectiveness

To enhance the likelihood of uptake if effective, the cost-effectiveness of 2 delivery models of YMHP in reducing sexual risk and substance use will be assessed utilizing CDC’s guidelines for cost-effectiveness analysis on HIV infections averted. The economic analysis will have 2 components: (1) a cost analysis of the YMHP intervention and (2) an incremental cost-effectiveness analysis that compares the value of clinic-delivery of YMHP over remote delivery. We will first estimate the marginal costs of delivering the 2 formats of YMHP. Using data from the modified Drug Abuse Treatment Cost Analysis Program [73,74] and study contact and expenditure records, key statistics from the cost evaluation will include the total annual economic cost for each program, weekly economic cost per client, and total economic cost per intervention session [75-77]. To highlight the relative contribution of the various cost components and necessary future budgeting, we will also perform a descriptive analysis of the cost accounted for by resource category.

The mean aggregate cost of the interventions will be used as inputs in the cost-effectiveness model. Cost-effectiveness will be modeled for clinic-delivery of YMHP as compared with remote-delivery for the differences in CAS and predicted through Markov modeling for 5 and 10 years and over a lifetime using varying assumptions about decay of the effect of the intervention over time. Modeling will be performed from the perspectives of (1) a third-party payer, (2) the medical care system, and (3) society.

#### Intervention Effects on Self-Management and Tests of Putative Mediation

Using GMM models similar to those described above in primary outcome analyses, we will examine the cross-time effects of intervention on the 5 dimensions of self-management. Where a significant between-condition difference in self-management is detected, we will explore mediation by examining whether intercept and/or growth factors for that dimension of self-management in turn predict outcomes. Indirect effects in GMM will be evaluated using bootstrapping estimation where possible. When this is not possible, a constraint approach will be employed. This approach involves comparing the fit of 2 models: one model in which the product of constituent pathways is constrained to be 0 and another in which the product of the direct effects is unconstrained. A significant Chi-square test associated with this comparison indicates that the constraint significantly diminishes model fit and constitutes evidence of the significance of the indirect pathway [78].

## Discussion

### Principal Findings

The goals of this YMHP intervention are to better understand HIV prevention–focused self-management behaviors among HIV-negative YMSM and to study the implementation of YMHP to improve portability and scalability. The SRVs will help to assess and address practical problems at the frontline of service provision to pave the way for a comprehensive program to reduce HIV infection among YMSM that reflects the complexities of real-world adolescent HIV clinics. If proven successful, this intervention delivery could help YMSM across the United States.

On the basis of previous studies, YMSM are at an even higher risk for HIV and STIs than older MSM [3,23,25]. YMSM living in urban areas and YMSM of color are especially at risk [6]. The need to lower these rates makes this study important. Similar interventions have been effective in the past. In a study of youth currently living with HIV, results showed that participants who had attended MI sessions were more likely to pursue behavior changes compared with participants in the control condition. The more sessions a participant attended, the better the results [5]. MI was also beneficial in a study of field outreach for young black men for HIV counseling and testing. The study found that outreach workers who had implemented MI were more likely to encourage youth to learn about their HIV status.

Implementing YMHP MI-based interventions targeting MSM at risk has been shown to be effective in past studies. This study is beneficial because it addresses the needs of the YMSM population. In urban areas, access to care (information about care or transportation means), PrEP information, and knowledge of sexual health and substance use might not occur in schools or with primary care providers; introducing all of this in a specially tailored intervention will give youth the information to lower their risk of HIV infection and STIs. It is important to implement this strategy drawing on the success of YMHP interventions in the past [79,80] because of the fact that YMSM as the population continues to see a rise in new HIV infection (132%), whereas rates among other groups have remained stable [6].

To address concerns related to differential drop out in that more remote delivery participants might never receive first session and that it is quite possible that retention across the 4 sessions is still higher in the remote condition because of transportation issues for in-person delivery, everyone will be asked to complete their session 1 in person regardless of their randomization to the intervention conditions. This would also address concerns some of the focus group youth had about developing a rapport over the phone or video chat. We estimate that retention for the IP and the 3-, 6-, 9-, and 12-month assessments will be 97%, 94%, 91%, 88%, and 85%, respectively, based on our previous study on YMHP [80,81]. Through REDCap, each clinic site will be able to generate reports for when YMSM are due for follow-up assessments, and the lead site, CHEST Hunter College, will provide extensive training to research assistants on retention efforts.

In addition, as YMHP is being tested in conjunction with other evidence-based practices in the Scale It Up program of research [58], intervention applicability and affordability will be determined through implementation science research methods and cost-effectiveness analysis. These components will be studied using EPIS to generate knowledge about the barriers and facilitators to the implementation and sustainment of the intervention into adolescent HIV prevention and clinical care settings.

### Limitations

This study has several possible limitations based on the population and locations involved. Another limitation regarding eligibility criteria is self-reporting of 3 or more days of substance use and 1 episode of CAS in the past 90 days. However, with the 3 SRVs HIV epicenters, intervention would be beneficial even as a preventative measure for this population. Although this study has a waiver of parental consent, parents or guardians might also come up as a barrier to participation. If parents do not approve of participation, they might discourage potential participants from participating or enrolled participants from completing the full study by refusing to cooperate if transportation to and from the clinic is dependent on the parent. Transportation overall might limit which participants can make it to appointments when they have to go to SRVs for testing, such as in Detroit where the lack of efficient public transportation is a barrier to participation in intervention studies. This is one of the main reasons why remote-based delivery is being tested in these real-world adolescent clinics.

## Abbreviations

ASSIST: Alcohol, Smoking and Substance Involvement Screening Test
ATN: Adolescent Medicine Trials Network for HIV/AIDS Interventions
BRIEF-A: Behavior Rating Inventory of Executive Function
CAS: condomless anal sex
CDC: Center for Disease Control and Prevention
CET: comparative effectiveness trial
CHEST: Center for HIV Educational Studies and Training
CHWs: community health workers
CT: Chlamydia trachomatis
EPIS: Exploration, Preparation, Implementation, Sustainment model
GC: Neisseria gonorrhea
GMM: growth mixture modeling
IP: immediate posttest
MI: motivational interviewing
MITI: Motivational Interviewing Treatment Integrity
MSM: men who have sex with men
NIH: National Institutes of Health
OMS: online master screener
PrEP: pre-exposure prophylaxis
REC: recruitment and retention center
SRVs: subject recruitment venues
STIs: sexually transmitted infections
YMHP: Young Men’s Health Project
YMSM: young men who have sex with men
